# Bacteremia Secondary to Epiploic Appendagitis (EA)

**DOI:** 10.7759/cureus.41648

**Published:** 2023-07-10

**Authors:** Daniella Abramov, Rachel Daniel, James R Pellegrini Jr., Ana P Rivera

**Affiliations:** 1 Plastic Surgery and General Surgery, New York Institute of Technology College of Osteopathic Medicine, New York, USA; 2 Internal Medicine, American University of the Caribbean School of Medicine, East Meadows, USA; 3 Internal Medicine, Nassau University Medical Center, East Meadow, USA

**Keywords:** conservative medical management, general surgery, sepsis, acute abdomen, epiploic appendagitis

## Abstract

Epiploic appendages are fat-filled sacs that are generally located along the surface of the large intestine. In most cases, epiploic appendagitis (EA) is described as an ischemic infarction of an epiploic appendage as the result of torsion or spontaneous thrombosis of the central draining vein of the epiploic appendage. The patient described in this report presented with a sudden onset of diffuse abdominal pain, nausea, and fever. CT scan of the abdomen and pelvis with oral contrast revealed EA of the sigmoid colon. Along the course of the admission, the patient became septic with blood cultures growing *E. coli*. In this case, we present a rare presentation of *E. coli* sepsis in the setting of EA, a usually uncomplicated and self-resolving presentation of abdominal pain.

## Introduction

Epiploic appendages are fat-filled pouches of the peritoneum that are most prevalent on the surfaces of the transverse and sigmoid colon [1]. The true function of epiploic appendages is unknown; however, it has been suggested that they have a physical role by serving as a cushion for the colon [2]. On average, the adult colon has approximately 50 to 100 appendages [3]. Epiploic appendages are freely mobile and consist of limited blood supply which makes them highly susceptible to torsion or thrombosis sometimes leading to ischemic or hemorrhagic infarction [2,3]. This is known as epiploic appendagitis. 

Epiploic appendagitis (EA) is a rare cause of abdominal pain, making up about 1.3% of abdominal pain presentations to the emergency department [4]. This may lead to misdiagnosis as other causes of abdominal pain such as appendicitis, cholecystitis, or ovarian torsion. Patients with EA typically experience sudden or gradual non-radiating dull constant lower abdominal pain, most commonly in the left lower quadrant but can also present in the right lower quadrant. Other, less common symptoms include early satiety, vomiting, bloating, diarrhea, and low-grade fever [5]. The majority of cases require symptomatic treatment and resolve within the course of several days. Therefore, it is important for patients to receive a proper workup in order to avoid misdiagnosis and unnecessary surgical intervention. We present a rare case of a patient with EA that progressed to bacteremia.

## Case presentation

A 54-year-old male with a past medical history of hyperlipidemia presented to the emergency department with complaints of sudden onset diffuse abdominal pain and right-sided back pain associated with fever and nausea. He denied experiencing similar symptoms in the past, vomiting, chest pain, dyspnea, dysuria, hematuria, decreased urine output, penile discharge, diarrhea, constipation, history of urinary tract infections, or nephrolithiasis. The rest of the review of systems was negative. The patient did not have any recent travel or sick contacts. 

**Figure 1:**
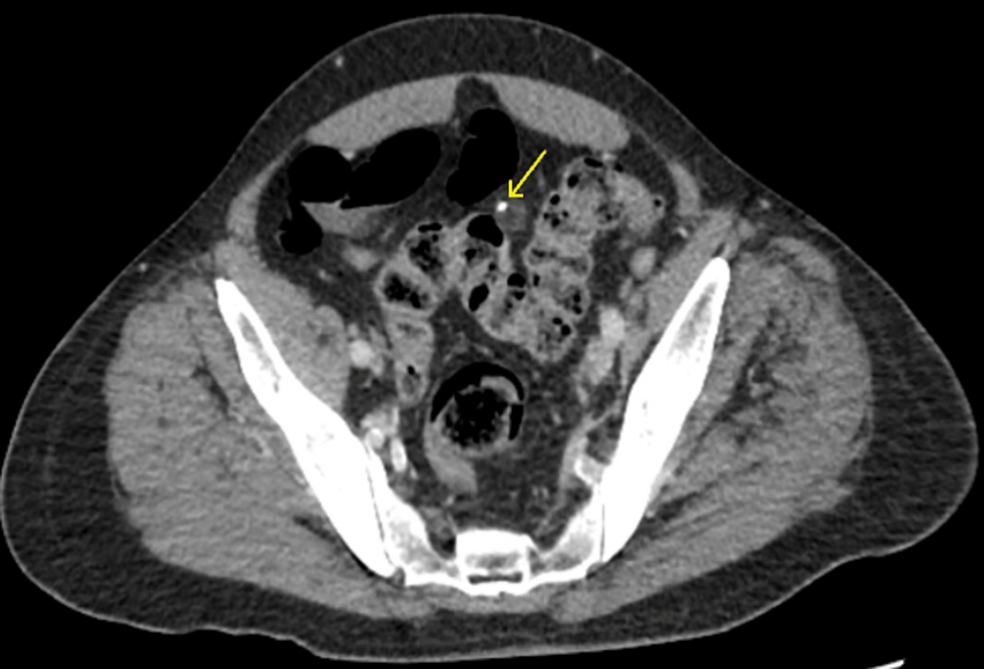
CT showing EA. The yellow arrow points to the involved epiploic appendage of the sigmoid colon. EA: Epiploic appendagitis

On physical exam, the patient appeared to be in moderate distress. Mucous membranes were dry. There was right costovertebral angle (CVA) tenderness. The abdomen was mildly distended and there was diffuse tenderness to palpation without rebound or guarding. There were no pulsatile or palpable masses on abdominal exam. The remainder of the physical exam was unremarkable. Vitals in the emergency department were: temperature of 103°F, blood pressure was 104/70 mmHg, heart rate was 138 bpm, and oxygen saturation was 95% on room air. The labs demonstrated elevated neutrophils and lactate levels. The results of the urinalysis, cardiac markers, electrocardiogram (EKG), and chest X-ray were unremarkable. A CT scan with contrast revealed EA (Figure 1).

The patient was admitted to the medical service for further workup blood cultures later demonstrated* E. coli* bacteremia. 

The patient continued to receive antibiotics for his septicemia. Surgery was consulted for the EA and recommended conservative management. After several days the patient clinically improved and was discharged home with instructions to follow up with his primary care physician and continue to take the antibiotics as indicated. 

## Discussion

EA was first described in the 1950s and can be divided into primary and secondary forms [2]. Primary EA is usually a result of inflammatory triggers secondary to torsion of the appendages or blockage or occlusion of the venous drainage. By contrast, and even rarer, secondary EA is usually a result of inflammation in structures near the epiploic appendages which include but are not limited to appendicitis, diverticulitis, postsurgical complications, and so forth.

Primary EA most commonly presents in male patients between their second and fifth decades of life, with a mean age of 40 years at diagnosis [6-8]. These demographics are consistent with the patient presented in this case. EA can arise in any segment of the colon and may be linked to risk factors such as obesity and recent significant weight loss, causing an increase in abdominal pressure, adipose tissue, and strenuous exercise [9]. In surgical case series, 57% of cases occur in the rectosigmoid, 26% in the ileocecum, 9% in the ascending colon, 6% in the transverse colon, and 2% in the descending colon [10,11]. 

In chronic EA due to gradual torsion of the appendages, patients are typically asymptomatic. If EA occurs with acute ischemia and infarction, this will result in fat necrosis which can be visualized on CT scan [12,13]. Acute EA results in non-specific symptomatic presentation of sudden onset lower abdominal pain associated with nausea, vomiting, diarrhea, and a low-grade fever. Currently, there are no specific lab findings indicative of EA. White blood cell count, erythrocyte sedimentation rate, and C-reactive protein levels are unremarkable or mildly elevated due to the inflammatory responses [2]. 

Since EA is a rare cause of abdominal pain and has non-specific symptoms and lab findings, it can be easily confused with other pathologies such as appendicitis, diverticulitis, pelvic inflammatory disease, and so forth [4-6]. It is essential to consider EA in the differential of abdominal pain since it is easily mistaken for other diagnoses that would be more likely to require surgical intervention. Reports indicate that EA is missed in 2% to 7% of patients with suspected acute diverticulitis and 0.3% to 1% of patients with suspected acute appendicitis [6]. 

The diagnosis of EA can be made using imaging such as CT scan or ultrasound with CT being preferred since it is more sensitive and specific as it confirms the fat necrosis as the result of EA [12]. The classic finding on CT scan is a 2- to 3-cm oval-shaped, fat-density, paracolic mass with thickened peritoneal lining and periappendageal fat stranding [11]. The fat density ovoid lesion and the central high attenuation focus within the fatty lesion are known as the ring sign and the dot sign, respectively [14]. On abdominal ultrasound, the inflamed appendage appears as a noncompressible, solid, hyperechoic ovoid mass with a subtle hypoechoic rim located at the point of maximal tenderness. Doppler studies typically reveal the absence of blood flow in the appendage in the setting of torsion but normal blood flow in the inflamed fat surrounding the appendage [15]. 

Once the diagnosis of EA is established with imaging, conservative pain management can be provided until symptoms resolve. However, according to the majority of cases, it is self-limiting. It is essential to monitor the patient for rare, but life-threatening complications such as abscess formation and/or septic shock. It has been proposed that ischemic and/or necrotic changes in the gut reduce the integrity of the tissue, allowing inflammatory cells and bacteria native to the gut to enter the bloodstream, leading to sepsis [16]. Sepsis management involves prompt identification, early initiation of antibiotics, and source control, along with hemodynamic support and organ function optimization. It requires close monitoring of vital signs, laboratory parameters, and timely interventions to mitigate the systemic inflammatory response and prevent organ dysfunction and failure [17].

## Conclusions

This case report emphasizes the importance of early recognition and appropriate management of EA to prevent its progression to sepsis, a potentially life-threatening complication. While EA typically resolves with symptomatic treatment, clinicians should remain vigilant for sepsis in these patients. Clinicians should maintain a high index of suspicion for sepsis in patients presenting with abdominal pain, especially in the context of an inflammatory condition like EA. Timely intervention is crucial as sepsis can lead to septic shock, organ failure, and increased mortality risk. The management of sepsis involves prompt control of the infection source through the administration of broad-spectrum intravenous antibiotics within the first hour of presentation, as well as fluid resuscitation to address shock. Prompt diagnosis, appropriate antibiotic therapy, and supportive care are essential for favorable outcomes in sepsis complicating EA. Continued research and awareness of the association between EA and sepsis are necessary to enhance clinical outcomes and patient safety. By maintaining a heightened awareness, healthcare professionals can effectively identify and manage this rare but serious complication, thereby improving patient outcomes and preventing potential complications.
